# Overexpression of a CPYC-Type Glutaredoxin, *OsGrxC2.2*, Causes Abnormal Embryos and an Increased Grain Weight in Rice

**DOI:** 10.3389/fpls.2019.00848

**Published:** 2019-06-27

**Authors:** Shengjie Liu, Hua Fu, Jieming Jiang, Zhongjian Chen, Jiadong Gao, Haoran Shu, Sheng Zhang, Chengwei Yang, Jun Liu

**Affiliations:** ^1^Agro-Biological Gene Research Center, Guangdong Academy of Agricultural Sciences, Guangzhou, China; ^2^Guangdong Provincial Key Laboratory of Biotechnology for Plant Development, College of Life Science, South China Normal University, Guangzhou, China; ^3^Rice Research Institute, Guangdong Academy of Agricultural Sciences, Guangzhou, China; ^4^Institute of Biotechnology, Cornell University, Ithaca, NY, United States

**Keywords:** embryogenesis, glutaredoxin (G*rxC2.2*), rice (*Oryza sativa* L.), seed, grain weight

## Abstract

Glutaredoxins (Grxs) are a ubiquitous group of oxidoreductase enzymes that are important in plant growth and development; however, the functions of rice Grxs have not been fully elucidated. In this paper, we showed that one of the Grxs, encoded by *OsGrxC2.2*, exhibited Grx activity. Furthermore, we demonstrated that *OsGrxC2.2* was able to regulate embryo development during embryogenesis. Transgenic rice lines overexpressing *OsGrxC2.2* unexpectedly exhibited degenerate embryos as well as embryoless seeds. Our data indicated that the embryonic abnormalities occurred at an early stage during embryogenesis. We found that the expression of several endodermal layer marker genes for embryo development, such as *OSH1* (apical region marker), *OsSCR* (L2 ground tissue marker), and *OsPNH1* (L3 vascular tissue marker), were significantly decreased in the *OsGrxC2.2-*overexpressed transgenic rice lines. In contrast, the transcript levels of the majority of protodermal layer markers, including *HAZ1*, *ROC2*, *ROC3*, and *RAmy1A*, and the shoot apical meristem marker *HB*, showed little change between the wild-type (WT) and *OsGrxC2.2*-overexpressing embryos. Surprisingly, the seed weight of the overexpressed transgenic rice was remarkably increased in comparison to that of the WT. These results indicate that the overexpression of *OsGrxC2.2* interferes with the normal embryogenesis of rice embryos and leads to increased grain weight. To the best of our knowledge, this is the first report that *OsGrxC2.2* is a rice embryo development-associated gene.

## Introduction

Rice (*Oryza sativa* L.) is the predominant staple food for more than half of the global human population. Although only accounting for 2–3% of the seed weight, the rice embryo is crucial for plant growth. Advancing our understanding of the molecular mechanisms of rice embryogenesis is important and could be used to improve grain weight and seed quality. The genetic control of embryogenesis has been studied via analyses of embryonic mutants and marker gene expression in rice (Hong et al., 1995; Kamiya et al., 2003; Yi et al., 2016). Rice mutants with an embryonic lethal phenotype have been identified and characterized, and several genes have been used as molecular markers for rice embryogenesis studies (Hong et al., 1995; Di Laurenzio et al., 1996; Kamiya et al., 2003; Ito et al., 2004). Despite these advancements in phenotypic identification, the molecular mechanisms of rice embryogenesis remain largely unknown.

Glutaredoxins (Grxs) are a ubiquitous group of oxidoreductases in both prokaryotes and eukaryotes (Holmgren, 1989; Holmgren and Aslund, 1995; Lillig et al., 2008). They are small glutathione-dependent oxidoreductases that belong to the thioredoxin (TRX) superfamily (El-Kereamy et al., 2015). Their predominant function is believed to be the reduction of disulfide bridges, and some members have recently also been shown to interact with iron-sulfur clusters (Riondet et al., 2012). Grxs can be divided into three major classes based on the predicted amino acid sequences and arrangement of the cysteine residues in the active-site motifs: CPYC-type, CGFS-type, and CC-type. The CC-type has only been identified in land plants, whereas the other two types have been documented in a range of organisms from prokaryotes to eukaryotes (Rouhier et al., 2004, 2006; Garg et al., 2010).

As a large gene family, the Grx family contains members with diverse roles. One of the most well-documented functions of Grxs in plants is their involvement in the oxidative stress response. They are implicated in various processes, such as in directly reducing peroxides or dehydroascorbate (DHA), reducing peroxiredoxins (Prxs), and also protecting the thiol groups on other enzymes via glutathionylation/deglutathionylation mechanisms (Rouhier et al., 2006). Both AtGRXcp (also termed AtGRXS14) and AtGRX4 (also termed AtGRXS15) play a pivotal role in protecting cells against oxidative stress (Cheng et al., 2006; Cheng, 2008). OsGRX8 in rice has also been implicated in the plant response to auxin, salinity, osmotic stress, and oxidative stress (Sharma et al., 2013).

Grxs can also regulate plant development by participating in DNA synthesis, signal transduction and stress responses, and [Fe–S] cluster aggregation (Rouhier et al., 2004, 2007, 2015). Recently, a few studies elucidated the roles of class I and class II GRXs in plant development. For instance, a CC-type glutaredoxin, OsGRX6, affects hormone and nitrogen status in rice plants, with its overexpression leading to a semi-dwarf phenotype (El-Kereamy et al., 2015). As for CPYC-type Grxs, AtGrxC1 and its homolog AtGrxC2 were found to be functionally redundant in dicotyledonous plants (Riondet et al., 2012). Knock-out mutants in *grxc1* or *grxc2* are aphenotypic, but the double mutant produces a lethal phenotype at an early stage after pollination, implying that GRXC1 and GRXC2 share redundant and vital functions (Riondet et al., 2012). In addition, the CGFS-type Grx AtGRXS17 (At4g04950) is critical in redox homeostasis and hormone perception where it mediates temperature-dependent postembryonic growth, with *grxs17* mutant plants showing abnormal apical meristems, elongated leaves, and impaired flowering under long photoperiods or at high temperature (Cheng et al., 2011; Knuesting et al., 2015).

The involvement of class III GRXs in the development of floral organs largely originates from genetic studies performed in *Arabidopsis thaliana* and *O. sativa*. In *A*. *thaliana*, the *roxy1* mutant exhibits a reduced number of petal primordia and abnormalities in petal morphogenesis (Xing et al., 2005), while the *roxy1 roxy2* double mutant is sterile and does not produce pollen (Xing and Zachgo, 2008). AtROXY4 was suggested to participate in gibberellin signaling and floral organ development. Indeed, plants overexpressing AtROXY4 displayed undeveloped petals and stamens and male sterility due to non-dehiscent anthers (Hou et al., 2008). In rice, the *mil1* mutant does not produce microspores in the anthers and is thus male-sterile, but female-fertile. The role of class III GRXs in the control of floral development notably relies on the redox regulation of TGA transcription factor activity, as shown primarily in *Arabidopsis* (Lee et al., 2006), and rice (Hong et al., 2012).

Although Grxs in rice have been known for decades, only limited information on their functions is available. Our previous study showed that *OsGrx* may protect embryo proteins from oxidation and thus might be correlated with seed storability (Gao et al., 2016). Another study demonstrated that *OsGrxC2.2*, a class I Grx isoform, is abundantly expressed in the aleurone layers in developing seeds and participates in oxidative stress defense in rice (Morita et al., 2015). We thus cloned *OsGrxC2.2* with the CPFC-type active site, a typical dithiol isoform, to investigate the specific roles of *OsGrxC2.2* in seed storage and protection from aging. We show that *OsGrxC2.2* overexpression in rice impairs embryo development and leads to increased grain weight.

## Materials and Methods

### Plant Materials and Growth Conditions

Wild-type rice Zhonghua11 (*O. sativa* ssp. *japonica*, WT) and transgenic lines seeds were cultivated in a growth chamber with a 28°C/26°C light/dark cycle of 14 h/10 h. The seedlings were transplanted under natural growing seasons into an experimental field of South China Normal University (Guangzhou, China).

### Generation of Transgenic Plants

The DNA fragments for fusing the promoter of *OsGrxC2.2* to β-glucuronidase (GUS) were constructed using a polymerase chain reaction (PCR)-mediated fusion strategy. The ∼2 kb promoter sequence of *OsGrxC2.2* was amplified using the primers listed in Supplementary Table S1. The PCR products were digested with *Sac*I and *Sal*I and inserted into the *pCambia1301* vector to generate the *OsGrxC2.2*promoter:GUS. Fifteen independent transgenic lines were selected for GUS activity analysis. To generate *OsGrxC2.2* overexpression lines, the full-length *OsGrxC2.2* cDNA from the WT was inserted into the plant binary vector *pCambia1390* using PCR-mediated gene fusion, as described above. Both constructs were transferred into the callus of the WT, using *Agrobacterium tumefaciens* strain EHA105. The resultant 12 independent rice lines were labeled OE1–OE12, respectively, and 4 of them (OE2, OE6, OE11, OE12) were used for the subsequent experiments.

### GUS Staining

For GUS staining, various tissues including four-leaf-stage root; tillering stage stem and leaf; heading stage flag leaf; a hull before flowering; flower; grain-filling-stage developing seeds (3–5, 10–12, and 15–17 days after flowering); and germinating seeds at 24 h, 36 h, 2 days, 3 days, 4 days, 5 days, and 6 days from the *pOsGrxC2.2:GUS* transgenic plants were incubated in a solution containing 50 mM sodium phosphate (NaPO_4_) buffer (pH 7.0), 5 mM potassium ferricyanide (K_3_Fe(CN)_6_), 5 mM potassium ferrocyanide (K_4_Fe(CN)_6_), 0.1% Triton X-100, 20% (v/v) methanol (MeOH), and 1 mM X-Gluc and stained at 37°C for 6 h and destained twice with 75% ethanol. They were then observed under a stereoscopic microscope SteREO Lumar V12 (Carl Zeiss, Heidenheim, Germany).

### 2,3,5-Triphenyltetrazolium Chloride (TTC) Assay

The TTC assay was performed as described by Wei et al. (2015), with some modifications. Hulled seeds were imbibed in distilled water for 12 h and then cut longitudinally into two identical halves. The cut seeds were incubated in freshly prepared 1× phosphate-buffered solution (PBS) containing 0.1% (w/v) TTC (Sigma-Aldrich, St. Louis, MO, United States) for 30 min at 37°C in a darkened incubator. Following incubation, the seeds were immediately rinsed with distilled water and washed twice with 75% (v/v) ethanol. The stained seeds were air-dried on filter paper and observed using a stereoscopic microscope.

### RNA Extraction and Quantitative Real-Time (qRT)-PCR

Total RNA was isolated from various tissues of the WT plants, including the root, stem, leaf, flag leaf, tiller, and panicle, and WT developing seeds (3, 6, 9, 12, 15, 18, and 21 days after pollination), and WT embryo and endosperm of the dry seeds, and 6 days embryos after pollination of the WT and transgenic rice lines using TRIzol reagent (Invitrogen, Carlsbad, CA, United States) according to the manufacturer’s instructions. Developing seeds were collected at 6 days after pollination (DAP), and the embryos were isolated under a microscope using a sterile blade and immediately placed in liquid nitrogen, following which they were stored at −80°C until analysis. The RNA samples were reverse-transcribed into first-strand cDNA using a PrimeScript RT Reagent Kit (Takara, Japan) and qRT-PCR reactions were carried out on a Bio-Rad Laboratories CFX Connect^TM^ Real-Time PCR Detection System with SYBR Premix Ex Taq (Takara, Japan). All experiments were conducted with at least three biological replicates. The qRT-PCR reactions were normalized based on the rice *Actin1* gene (*OsActin1*), which was used as an internal control using the 2^–ΔΔ^*^C^*^t^ method (Livak and Schmittgen, 2001). The primers designed by Primer Premier 5.0 software for the analysis of transcript levels are listed in Supplementary Table S1.

### Detection of the Subcellular Localization of *OsGrxC2.2*

To verify the subcellular localization of *OsGrxC2.2*, the yellow fluorescent protein (YFP) gene was fused in-frame to the C-terminal of OsGrxC2.2 into the pSAT6-EYFP vector to generate 35S:*OsGrxC2.2*-*YFP*, where the fusion gene was expressed under the control of the cauliflower mosaic virus 35S promoter.

The plasmid was transformed into rice protoplasts prepared from the leaf sheaths of 10-days-old seedlings. Rice protoplast transformation was performed according to previously reported methods (Yoo et al., 2007; Zhang et al., 2011). The YFP signal of the OsGrxC2.2-YFP fusion protein was visualized under a confocal microscope (LSM710, Carl Zeiss, Heidenheim, Germany).

### Expression, Purification, and Enzymatic Characterization of OsGrxC2.2

To express the OsGrxC2.2 protein in prokaryotic cells, *OsGrxC2.2* was cloned into the expression vector PET-28a to construct the *OsGrxC2.2*-PET-28a recombinant plasmid, which was transformed into the BL21 (DE3) *Escherichia coli* strain. Expression and purification of recombinant proteins were performed as previously reported (Chi et al., 2012), with minor modifications. The recombinant *OsGrxC2.2* was expressed as a His_6_-tagged fusion protein and purified using affinity chromatography with nickel-chelating Sepharose (Sigma-Aldrich). Fifteen microliters of each fraction was loaded into each lane of a 15% SDS-PAGE gel, followed by Coomassie brilliant blue R-250 staining.

The purified OsGrxC2.2 activity was determined using the β-hydroxyethyl disulfide (HED) assay (Chi et al., 2012; Riondet et al., 2012). The reaction mixture consisted of 100 mM Tris-Cl (pH 7.4), 3 μg glutathione reductase (GR; Sigma-Aldrich), 0.75 mM HED, 0.2 mM nicotinamide adenine dinucleotide phosphate (NADPH; Sigma-Aldrich), and 1 mM glutathione (GSH, Sigma-Aldrich) in a final volume of 100 μL. A mixed disulfide between HED and GSH was formed within 2 min, and the reaction was initiated by the addition of 0.06–0.40 μg OsGrxC2.2 and was followed by a decrease in absorbance at A_340_ due to the oxidation of NADPH. The control reaction included all the reagents except OsGrxC2.2.

### Histochemical Analyses

The developing seeds at various stages after pollination, including 3, 5, 7, and 10 days, were fixed with 3% (w/v) paraformaldehyde and dehydrated in a graded ethanol with series of 75, 85, 90, 95, and 100%. The samples were embedded in paraplast and then sectioned into 8 μm-thick sections, stained with 0.1% toluidine blue, and observed with a Zeiss Axio Scope A1 microscope (Carl Zeiss, Heidenheim, Germany).

### Measurement of Rice Seed Weight

The grain weight was estimated using a 1000 seeds of the transgenic lines and WT in three biological replicates; the seed weight was estimated using a hundred dehulled seeds of the transgenic lines and WT in three biological replicates. The data were presented as mean ± standard error (SE).

### Data Analysis

Three-sample *t*-tests or one-way analysis of variance (ANOVA), in conjunction with LSD tests, were used to identify significant differences at *p* < 0.05 or *p* < 0.01.

## Results

### Expression Pattern and Subcellular Localization of OsGrxC2.2

**FIGURE 1:**
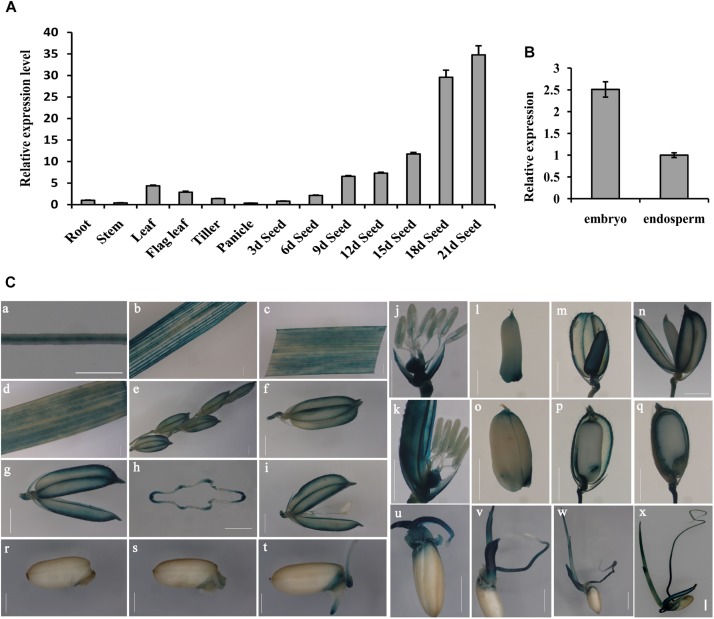
Expression pattern analysis of *OsGrxC2.2*. **(A)** Measurement of *OsGrxC2.2* gene expression in various rice tissues. **(B)** Comparison of *OsGrxC2.2* gene expression in the embryo and endosperm from dry seeds. The rice *Actin1* gene (*OsActin1*) was used as an internal control. **(C)** Histochemical analysis of GUS activity in *pOsGrxC2.2*:GUS plants. Four-leaf-stage root **(a)**; tillering stage stem **(b)**, leaf **(c)**; heading stage flag leaf **(d)**, a hull before flowering **(e–h)**, flower **(i–k)**; grain-filling stage developing seeds of 3–5 days **(l–n)**, 10–12 days (o, p), and 15–17 days **(q)** after flowering; and germinating seeds of 24 h **(r)**, 36 h **(s)**, 2 days **(t)**, 3 days **(u)**, 4 days **(v)**, 5 days **(w)**, and 6 days **(x)**; **(h)** is the cross section of **(g)**. Bar = 0.5 cm.

To determine the *OsGrxC2.2* expression pattern, total RNA from various tissues and organs was extracted. RT-PCR using *OsActin1* as an internal control, was performed as described in the Materials and Methods. In all the examined tissues and organs, *OsGrxC2.2* was found to be highly expressed in the leaf, flag leaf, and developing seeds at different stages compared to the root, stem, tiller, and panicle (Figure 1A). Interestingly, the expression of *OsGrxC2.2* was even higher in the developing seeds, particularly in the late stages (18 and 21 DAP). The expression was also much higher in the embryo than in the endosperm (Figure 1B), suggesting that the OsGrxC2.2 enzyme may be involved in the developing seeds and embryos.

The YFP signal of the OsGrxC2.2-YFP fusion protein was visualized under a confocal microscope, which demonstrated that the OsGrxC2.2 protein was expressed and localized in the cytoplasm (Supplementary Figure S1). To elucidate the spatiotemporal expression profiles of *OsGrxC2.2* in rice, the *OsGrxC2.2* promoter was fused to the GUS reporter gene and transformed into WT plants. The GUS activity was detected in most of the examined tissues and organs, including the roots, culms, leaves, flowers, young spikelets, and developing caryopses at various stages. High GUS activity was measured in the vascular bundles of the roots, stems, leaf blades, hulls, caryopses, and filaments (Figures 1C,a–k). Additionally, high activity was also observed in various regions of the developing seeds, particularly in the embryos and outer layers of the endosperm, including the aleurone and subaleurone tissues (Figures 1C,l–q). GUS staining analysis of the germinating seeds (1–6 days) indicated that the activity of the promoter of *OsGrxC2.2* was stronger in the embryo, coleorhizae, coleoptiles, and young leaf blades (Figures 1C,r–x). As shown in Figure 1C, relatively weak GUS activity was noted in the young leaf blades and blade sheaths.

### Expression, Purification, and Enzymatic Characterization of OsGrxC2.2

**FIGURE 2:**
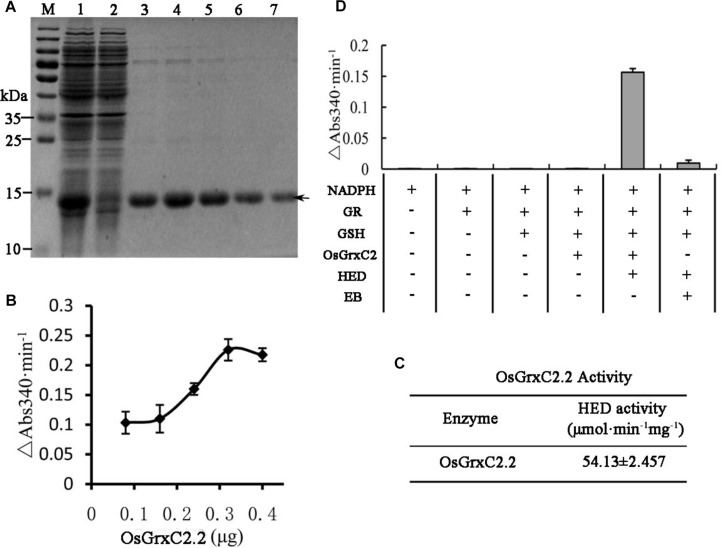
Expression, purification, and enzymatic characterization of OsGrxC2.2. **(A)** Expression and purification of recombinant OsGrxC2.2 in *E. coli.* Fifteen microliters of each fraction was loaded into each lane of a 15% SDS-PAGE gel, followed by Coomassie brilliant blue R-250 staining. Lane 1, crude extracts from *E. coli* expressing OsGrxC2.2; lane 2, flow-through proteins from the Ni-NTA column; lanes 3–7, OsGrxC2.2 eluted from the Ni-NTA column. Molecular masses (in kDa) of standards are shown on the left. The arrow indicates the target protein. **(B)** Determination of OsGrxC2.2 protein enzymatic activity followed by NADPH oxidation rate. NADPH oxidation was monitored at 340 nm in a 100 μL reaction mixture containing 0.1 M Tris-HCl (pH 7.4), 0.6 μg GR, 0.75 mM HED, 0.2 mM NADPH, and OsGrxC2.2 at concentrations varying from 0.06 to 0.3 μg. The rate of NADPH oxidation is shown by △Abs340min^–1^. **(C)** Enzymatic activity of OsGrxC2.2. **(D)** Effects of various combinations of GR, GSH, HED, EB (elution buffer), and OsGrxC2.2 on the initial rates of NADPH oxidation.

In order to evaluate the enzymatic characteristics of OsGrxC2.2, the coding region of *OsGrxC2.2* (384 bp) was amplified by PCR and subcloned in an expression vector, pET-28a (+), as described in the Materials and Methods, and overexpressed in *E. coli*. An SDS-PAGE image for the *E. coli* lysate showed an over-expressed 14.5 kD protein (Figure 2A). As expected, the corresponding gel band was identified and confirmed to be OsGrxC2.2 by matrix-assisted laser desorption/ionization (MALDI) mass spectrometry. An HED assay was then used to examine the Grx activity of OsGrxC2.2. The results show that the purified OsGrxC2.2 enabled the reduction of the disulfide bond of HED with specific activity at 54.1 (μmol/min/mg) (Figures 2B,C). Although weak NADPH oxidation activity was also observed in elution buffer (EB), it was negligible in comparison to that of OsGrxC2.2 (Figure 2D).

### Abnormal Embryo Phenotypes of Transgenic Rice With Overexpressed *OsGrxC2.2*

**FIGURE 3:**
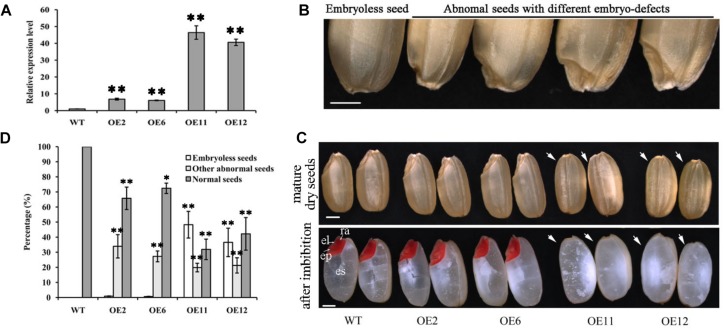
Phenotypic analysis of seeds overexpressing *OsGrxC2.2*. **(A)** qRT-PCR analysis of *OsGrxC2.2* expression in the four independent transgenic lines OE2, OE6, OE11, and OE12. **(B)** Seeds with different types of embryo. **(C)** Embryo phenotype of the WT and *OsGrxC2.2* overexpression lines. Upper panel, mature embryos from dry seeds; lower panel, TTC staining of rice seeds with imbibition at 12 h. cp, coleoptiles; el, embryonic leaves; ra, radical; arrow, embryos. Bars = 1 mm. **(D)** Percentage of different types of embryos in the WT seeds and *OsGrxC2.2*-overexpression line seeds. Error bars show the standard deviation. Statistical significance is indicated by ^∗∗^*p* < 0.01 and ^*^p < 0.05.

To further characterize the potential influence of *OsGrxC2.2* on rice growth and development, transgenic rice lines overexpressing *OsGrxC2.2* under the Ubi promoter (*pUbi::OsGrxC2.2*) were constructed. As indicated in Figure 3A, there were four representative overexpression lines demonstrating significant up-regulation of *OsGrxC2.2* compared to the WT rice. Furthermore, the seeds obtained from the four homozygous transgenic lines revealed degraded and/or aborted embryos (Figure 3B). The overexpression lines OE11 and OE12 exhibited the most significant *OsGrxC2.2* up-regulation compared with the OE2 and OE6 lines (Figure 3A). Accordingly, lines OE11 and OE12 possessed considerably more abnormal embryos, such as organless and radicleless embryos and displayed a more obvious embryoless seed phenotype than observed in OE2 and OE6 (Figure 3C). As indicated in Figure 3D, there was an average of approximately 60% abnormal embryo seeds in lines OE11 and OE12 (39% embryoless seeds plus 21% other abnormal seeds), while this value was 31% in lines OE2 and OE6 (1% embryoless seeds plus 30% other abnormal seeds).

### Abnormal Embryos Appeared at an Early Stage of Embryogenesis

**FIGURE 4:**
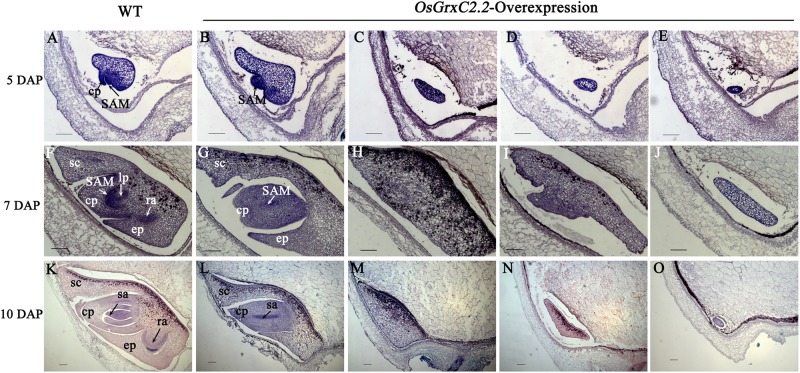
Median longitudinal sections of WT and *OsGrxC2.2*-overexpression lines embryos at various developmental stages. Developing embryos of WT **(A,F,K)** and representative overexpression line OE11 **(B–E,G–J,L–O)** at 5 DAP **(A–E)**, 7 DAP **(F–J)**, and 10 DAP **(K–O)**. cp, coleoptiles; ep, epiblast; lp, leaf primordium; ra, radical apex; sa, shoot apex; SAM, shoot apical meristem; sc, scutellum are as indicated. Bars = 100 μm.

To determine when the embryonic defects occurred in the overexpression lines, the developing embryos of the transgenic lines were subjected to histochemical analysis. At 3 DAP, a similar globular embryo was formed in the zygote in both the WT and overexpression lines (Supplementary Figure S2). At 5 DAP, the coleoptile primordium began to differentiate in the WT, and the shoot apical meristem (SAM) was recognizable as a bulging protrusion at the base (Figure 4A). However, some embryos in the overexpression lines differed from those of the WT at this stage and appeared to form abnormal embryos, including an elliptic curved embryo, spindle embryo, minor embryo, and other embryo types (Figures 4B–E). Consequently, some embryos failed to develop organs, including the coleoptile primordium and the SAM. At 7 DAP, the primordium of the first leaf in the WT emerged below the shoot apex, the radicle was formed, and the coleoptile and SAM were elongated (Figure 4F). In contrast, some abnormal embryos in the overexpression lines showed little differentiation from the radicleless embryos, sickle-shape embryos, “T”-shaped embryos, and the oval germinal embryos (Figures 4G–J). By 10 DAP, all organs had developed in the WT and embryogenesis was complete (Figure 4K), with the seeds continuing to develop until maturation. On the contrary, neither growth nor differentiation was noted in the abnormal embryos of the overexpression lines (Figures 4M–O), which constituted abnormal seeds or embryoless seeds that failed to germinate.

### Expression of Embryogenesis Marker Genes in *OsGrxC2.2* Overexpression Lines

To investigate the molecular mechanisms underlying the abnormal embryos, we isolated embryos from the overexpression lines and WT at 6 DAP and determined the transcript level of *OsGrxC2.2* and several representative embryogenesis-related genes.

**FIGURE 5:**
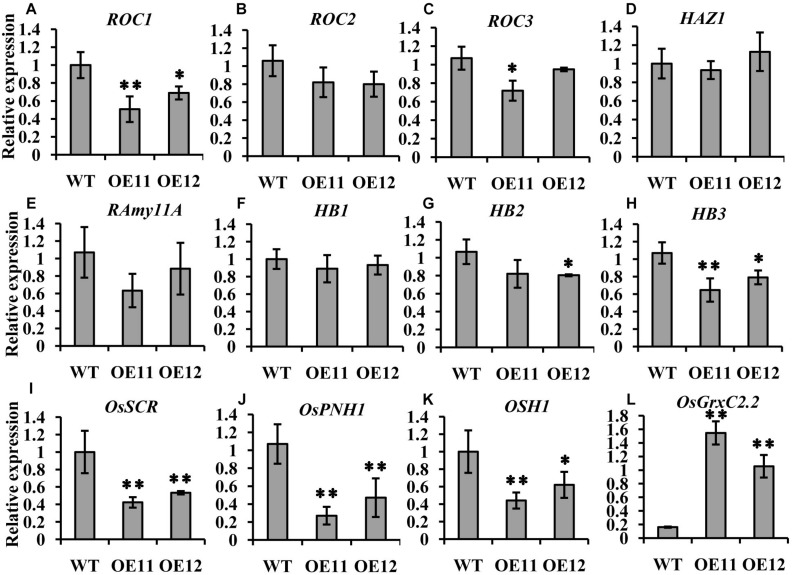
Expression comparison of molecular marker genes between the *OsGrxC2.2*-overexpression lines (OE11 and OE12) and the WT control. qRT-PCR of *ROC1* **(A)**, *ROC2* **(B)**, *ROC3* **(C)**, *HAZ1* **(D)**, *RAmy11A* **(E)**, *HB1* **(F)**, *HB2* **(G)**, *HB3* **(H)**, *OsSCR* **(I)**, *OsPNH1* **(J)**, *OSH1* **(K)**, and *OsGrxC2.2* **(L)**. The *y*-axis represents the gene expression relative to the *OsActin1* transcript level. Results are the averages of three independent experiments. Error bars show the standard deviation. Statistical significance is indicated by ^∗∗^*p* < 0.01 and ^*^p < 0.05.

Among the three *ROCs*, which are markers for the L1 layer, only *ROC1* showed relatively lower expression levels in the overexpression lines embryos, while the expression of *ROC2* and *ROC3* was the same as that in the WT (Figures 5A–C). The transcript level of the outer layer gene *HAZ1* was similar between the overexpression lines and the WT (Figure 5D). Furthermore, no impact on the expression of the L1 epithelium layer marker *RAmy1A* and the SAM markers *HB1* and *HB2* was observed. *HB3* was decreased in the overexpression line embryos (Figures 5E–H).

Interestingly, two other markers, *OsSCR* at the L2 ground tissue and *OsPNH1* at the L3 vascular tissue were significantly changed. The transcript levels of *OsSCR* and *OsPNH1* were dramatically decreased in the overexpression line embryos (Figures 5I,J). The expression of *OSH1*, a marker of the apical region, was also considerably decreased in the overexpression lines embryos (Figure 5K). However, the transcript level of *OsGrxC2.2* was significantly higher in the overexpression lines than in the WT (Figure 5L). These results are consistent with the abnormal embryo phenotypes observed by histochemical analysis (Figure 4), which demonstrated that *OsGrxC2.2* is involved in negatively regulating the differentiation of the embryonic organs and the basic axis during embryogenesis.

### Impact of *OsGrxC2.2* Overexpression on Endosperm Development and Seed Weight

Although embryo development was affected by *OsGrxC2.2*, the seed length and hull color of the seeds in the overexpression lines did not differ from that of the WT (Supplementary Figure S3). To further investigate whether *OsGrxC2.2* affects endosperm development, we analyzed the transcripts of the genes involved in starch synthesis in the caryopses at 10 DAP, the time point when starch is being actively accumulated (Yamakawa et al., 2007). We found that the expression levels of starch biosynthesis genes encoding ADP-glucose pyrophosphorylase large subunit 1 (AGPL1), Starch synthase IIIa (SSIIIa), and Starch branching enzyme I (BEI) did not differ between the WT and transgenic lines (Supplementary Figure S4). The expression of a starch debranching enzyme gene, *pullulanase*, in the overexpression lines was slightly higher than that in the WT seeds (Supplementary Figure S4), which corroborates previous studies (Ohdan et al., 2005; Lee et al., 2015).

**FIGURE 6:**
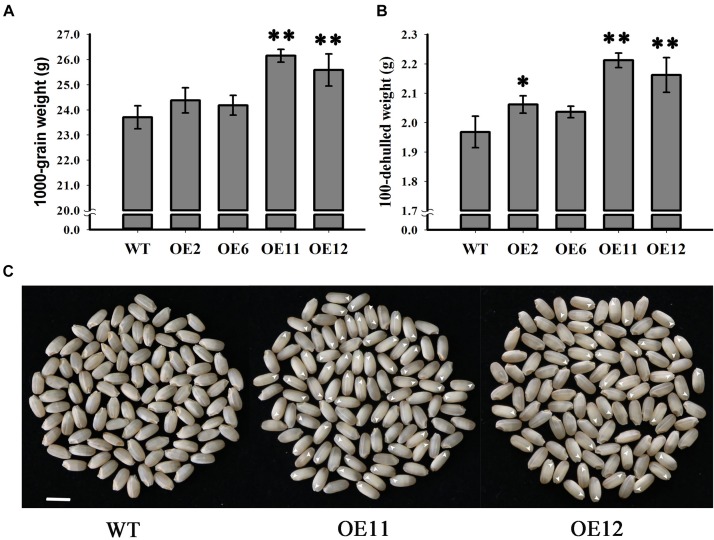
Analysis of grain weight in WT and *OsGrxC2.2*-Overexpression lines. **(A)** 1000-grain and **(B)** 100-seed weight of WT and *OsGrxC2.2* overexpression line (OE2, OE6, OE11, and OE12) seeds. **(C)** Phenotypical comparison of seeds from WT and the *OsGrxC2.2* overexpression plants. Bar = 0.5 cm. Arrows: embryoless seeds. Statistical significance is indicated by ^∗∗^*p* < 0.01 and ^*^p < 0.05.

Since seed weight is an important indicator of crop yield, the seed weights from the overexpression lines and the WT plants were determined and compared. Statistical analysis indicated that the seed weights of the overexpression lines OE11 and OE12 were very significantly greater than those of the OE2, OE6 and WT (*p* < 0.01) (Figure 6A). The 1000-grain weight of lines OE11 and OE12 were 26.15 and 25.58 g, respectively, while the 1000-grain weight of the WT was 23.7 g. However, the 1000-grain weights of lines OE2 and OE6 were 24.37 and 24.18 g, respectively, which is higher but not significantly different from that of the WT. A similar trend is presented in Figure 6B about the dehulled seed weight. As the overexpression lines OE11 and OE12 possessed many more embryoless seed (39%) (Figure 6C) than in OE2 and OE6 (1%) (Figure 3D), the results suggest that the contribution to the increased seed weight mainly comes from the embryoless seeds.

## Discussion

### *OsGrxC2.2* Interferes With the Normal Embryogenesis of Rice Seed Development

Although plant genomes contain many *Grxs* (Lemaire, 2004; Rouhier et al., 2006), only a few have been characterized (Rouhier et al., 2004). Garg et al. (2010) identified 48 genes encoding Grx proteins in the rice genome and suggested diverse roles for these *Grx* genes during growth and development. Some of the *Grx* genes were only expressed in specific organs/developmental stages, and the expression of many rice *Grx* genes was modulated by various phytohormones and abiotic and biotic stress conditions, suggesting an important role for *Grx* proteins in response to these stimuli (Garg et al., 2010). For instance, *OsGRX8* in rice has been implicated in the plant response to stress (Sharma et al., 2013). However, the functions of most of these *Grx* genes are unclear.

In our previous study, several redox regulation proteins, mainly glutathione-related proteins including one Grx protein (CAA54397.1), exhibited various degrees of change in protein abundance during storage between hybrid rice seeds with different storability, and thus might play an important role in protecting embryo proteins from oxidation (Gao et al., 2016). Another study reported that the OsGrxC2.2 protein accumulates abundantly in the embryo and aleurone layers, and participates in oxidative stress defense in developing and mature seeds (Morita et al., 2015).

Given these findings, our original intention was to explore the function of *OsGrxC2.2* in seed stress resistance. We thus cloned the Class I *Grx* isoform *OsGrxC2.2* (Os04g42930) with a CPFC active site, which is a typical dithiol isoform (Supplementary Figure S5). However, we unexpectedly discovered that *OsGrxC2.2* overexpression induces embryo malformation or abortion (Figure 3B). This finding motivated us to redirect our research to investigate the roles of the OsGrxC2.2 protein in embryonic development and explore the possible associated mechanisms.

Grxs have recently been found to play an important role in plant developmental processes (Rouhier et al., 2015). The overexpression of *OsGRX6* led to a semi-dwarf phenotype (El-Kereamy et al., 2015), while OsMIL1 is involved in the floral organ development (Hong et al., 2012). A previous study reported the *OsGrxC2.2* homologous gene *AtGrxC2* in *A. thaliana*, found that the double *grxc1 grxc2* mutant is lethal. The most likely hypothesis is that a very early stage of seed development is affected in the double *grxc1 grxc2* mutant (Riondet et al., 2012), but no such reports on the equivalent *OsGrxC2.2* were found in rice. Since rice is a crop plant, the unique role of *OsGrxC2.2* in rice embryonic development could have significant economic implications for grain production. We thus speculated that *OsGrxC2.2* might be crucial in seed maturation and particularly involved in embryo development.

Our histochemical assay revealed a higher level of GUS activity (representing OsGrxC2.2 localization as a fusion protein) in the vascular tissues (including the root, stem, leaf, and filament), embryo, and aleurone layer (Figure 1B). This corroborates a previous study by Garg et al. (2010), which demonstrated that *OsGrxC2.2* mRNA was expressed at a high level in various tissues, including the leaves, roots, and developing seeds in comparison to other CPFC-type Grxs in rice. However, our findings differ from those of Morita et al. (2015) in that *Grx* expression was not detected in the leaf in their study. This inconsistency might be related to post-transcriptional regulation, where a number of genes are transcribed but cannot be translated into proteins in certain tissues due to their modification.

In addition, the qRT-PCR data did not well match with those from the GUS reporter system, for instance in the leaf. Studies have shown that sequences in the intron and/or 3’UTR can regulate gene transcription. We speculate that additional regulatory regions might exist in the intron and/or 3’UTR of *OsGrxC2.2*. Alternatively, we also cannot exclude the possibility that the promoter region we used was not long enough, thus resulting in some regulatory sequences being missed. Future studies are required to test these possibilities.

### *OsGrxC2.2* Overexpressing Rice Lines Showed Degenerate Embryoless Phenotypes

During the early stages of embryogenesis, a rice embryo first establishes three axes: apical–basal, radial, and dorsal–ventral (Sato, 2008; Yi et al., 2016). Along the apical–basal axis, the SAM, cotyledon, hypocotyls, and root apical meristem (RAM) form, while the radial axis consists of the epidermis (L1 layer), ground tissue (L2 layer), and central vascular cylinder (L3 layer) form perpendicular to the apical–basal axis, and develop from the outside in Mayer et al. (1991), Kamiya et al. (2003), and Yi et al. (2016).

To identify the point at which the morphological distinction appeared during embryogenesis, we conducted a precise histological analysis of the overexpression lines and WT embryos. Rice embryos complete their morphogenic events within 9–10 days under normal conditions, and the globular stage, which does not exhibit any morphological organ differentiation, continues until 3 DAP (Sato et al., 1996; Ito et al., 2002). No significant difference was found between the overexpression lines and WT embryos at the globular stage. However, from 5 DAP, the overexpression lines embryos began to differ from those of the WT, in that some did not undergo differentiation of the stem-tip tissues (Figures 4C–E), or otherwise displayed abnormal embryos, including organless embryos (Figures 4H–J,M–O) and radicleless embryos (Figures 4G,L).

Following this, we measured the expression of embryo development-related marker genes in the *OsGrxC2.2*-overexpressed rice lines. Several genes that are expressed in specific cell types and regions have been used as molecular markers in rice embryogenesis studies. For example, the rice *SCARECROW (OsSCR)* is specifically localized in the cortex/endodermis cell layer that corresponds to the L2 layer (Di Laurenzio et al., 1996); the rice *PINHEAD/ZWILLE* gene *(OsPNH1)* is expressed in the vascular regions of the leaf primordia and is used as an L3 vascular tissue marker (Nishimura et al., 2002; Kamiya et al., 2003); and *OSH1* is expressed in the indeterminate cells around the SAM and is used as a marker for the apical region in rice embryos (Kamiya et al., 2003; Sato, 2008). Other developed markers include Rice outermost cell-specific gene (*ROC*, marker for the L1 layer) (Ito et al., 2002), Rice α-amylase1 (*RAmy1A*) (marker for the L1 layer of the epithelium) (Sugimoto et al., 1998), and *OSH1* (marker for the apical region) (Sato et al., 1996).

The expression levels of most of the outer layers were similar between the overexpression lines and the WT (Figure 5).

However, the expression of *OSH1* was significantly decreased in the overexpression line embryos (Figure 5K). Furthermore, the transcript levels of *OsSCR* and *OsPNH1* were also decreased in the overexpression line embryos (Figures 5I,J). The significant decrease in the transcript levels of *OsSCR*, *OsPNH1*, and *OSH1* in the central region indicated that differentiation in the ground tissue and vascular primordium had been affected. Importantly, the overexpression lines were partly defective in the establishment of the L2 and L3 layers from the inner cell mass.

Matching conversely with these marker genes in the central region, the expression of *OsGrxC2.2* was significantly higher in the overexpression lines (Figure 5L). *OsGrxC2.2* overexpression appears to be involved in rice embryogenesis, and negatively affects embryo development, resulting in embryo degeneration or abortion, or even the formation of embryoless seeds (Figure 3) by reducing the expression of several L2 and L3 marker genes associated with embryo development. However, it is unclear whether the overexpression of *OsGrxC2.2* directly reduces the expression of several L2 and L3 marker genes, or whether another yet unknown redox-based process leads to defects in embryo L2 and L3 differentiation and a reduction of marker genes expression. Further research is required to clarify these possibilities.

### Overexpression of *OsGrxC2.2* Influences Rice Seed Weight

In the present study, we demonstrated that the overexpression of *OsGrxC2.2* in rice impacts embryo development, resulting in seeds without embryos or with a smaller embryo, with the seed embryos being completely or partially replaced by endosperms. The increased weight of the *OsGrxC2.2*-overexpressing seeds can probably be attributed to the large endosperm, which may occupy the entire space in some instances (Figure 6B). This mechanism differs from that of *OsGRX6*, which affects hormone signaling and nitrogen status in rice plants leading to a semi-dwarf phenotype and delayed chlorophyll degradation (El-Kereamy et al., 2015).

Grxs possess peroxidase activity and the capability to reduce DHA and type II Prxs, regenerate methionine sulfoxide reductase, and interact with thioredoxins. Therefore, they play important roles in various cellular processes, including the maintenance and regulation of cellular redox state, iron homeostasis, and redox-dependent signaling pathways, which eventually contribute to the removal of reactive oxygen species (ROS) and the repair of lipid and protein oxidative damage (Wells et al., 1990; Sha et al., 1997; Rouhier et al., 2001). Furthermore, there is growing evidence suggesting that ROS-mediated redox signals (redox homeostasis) function during plant development and adaptation to stress conditions, such as those occurring during seed germination, root hair development, stomatal closure, and root gravitropic responses (Joo et al., 2001; Foreman et al., 2003; Gapper and Dolan, 2006; Kwak et al., 2006; Achard et al., 2008). Therefore, we speculate that OsGrxC2 might be crucial in seed embryo development, and alters normal embryogenesis through interfering with redox homeostasis in the early rice embryo, thus leading to increased grain weight. We believe that our observation could be further explored in future studies to improve grain weight and yield using biotechnology.

In higher plants, embryo and endosperm originate from double fertilization. Rice embryos contain grass-specific coleoptiles, scutella, and epiblasts, suggesting that there are more complicated molecular mechanisms controlling rice embryo development. As the rice endosperm grows continuously and occupies most of the space in the mature seed, the developmental coordination of the embryo and endosperm is of particular importance for the proper development of rice seeds. However, the associated molecular pathways remain poorly understood. It has been suggested that the endosperm and embryo in rice modulate one another’s growth due to space limitations (Hong et al., 1996). Indeed, temperature-sensitive *embryoless1* mutants produce embryoless seeds with large endosperm at high temperature, but display large embryos with small or even no endosperm at low temperature (Hong et al., 1995). The down-regulation of *Orysa;CycB1;1* resulted in severe endosperm defects, but also resulted in the production of very large embryos (Guo et al., 2010). In our study, the overexpression lines OE11 and OE12 exhibited significantly higher *OsGrxC2.2* level than the OE2 and OE6 lines (Figure 3A), and possessed much more embryoless seeds than those observed in OE2 and OE6 (Figure 3C). Consequently, both OE11 and OE12 display a significantly higher weight than that of the OE2 and OE6, and WT. This result suggests that *OsGrxC2.2* overexpression impairs embryo development, and subsequently leads to increased endosperm size and seed weight.

In summary, we found that transgenic rice overexpressing *OsGrxC2.2* resulted in a novel phenotype with degenerate embryos and even embryoless seeds. The embryonic abnormalities began at the late globular stage during embryogenesis. In addition, the grain weight of the overexpression lines was significantly increased in comparison to the WT. These results indicate that overexpression of *OsGrxC2.2* interferes with the normal embryogenesis and leads to increased grain weight.

## Data Availability

All datasets generated for this study are included in the manuscript and/or the Supplementary Files.

## Author Contributions

SL, CY, and JL conceived the project and designed the research. SL, HF, JJ, HS, and JG performed the research. SL, HF, ZC, SZ, CY, and JL analyzed the data. SL and JL wrote the manuscript. All authors read and approved the final version of the manuscript.

## Conflict of Interest Statement

The authors declare that the research was conducted in the absence of any commercial or financial relationships that could be construed as a potential conflict of interest.

## Supplementary Material

FIGURE S1 Subcellular localization of the OsGrxC2.2 protein.

FIGURE S2 Median longitudinal sections of the WT and *OsGrxC2.2*-over expression lines embryos at 3 DAP. Developing embryos of WT **(A)** and representative overexpression line OE11 **(B)**.

FIGURE S3 Phenotypic analysis of embryoless and WT seeds. **(A)** Hulled seeds. **(B)** Shelled embryoless and WT seeds.

FIGURE S4 Analysis of endosperm development maker genes in WT and *OsGrxC2.2*-overexpression lines. Expression levels of genes involved in starch biosynthesis obtained via qRT-PCR analyses of *AGPL1*, *OsSSIIa*, *BE1*, and *PUL* from 10 DAP seeds of WT and *OsGrxC2.2*-overexpression lines. The *y*-axis represents the gene expression relative to the *OsActin1* transcript level. Results are the averages of three independent experiments.

FIGURE S5 Amino acid sequence alignment between OsGrxC2.2 and other class I Grx sequences in rice using ClustalW software.

TABLE S1 Primer sequences used in this study.
