# Supplementary figures and images for: Overexpression of a CPYC-Type Glutaredoxin, OsGrxC2.2, Causes Abnormal Embryos and an Increased Grain Weight in Rice
Source: Front Plant Sci. 2019 Jun 27;10:848. doi: 10.3389/fpls.2019.00848 (PMC6610441; doi:10.3389/fpls.2019.00848)

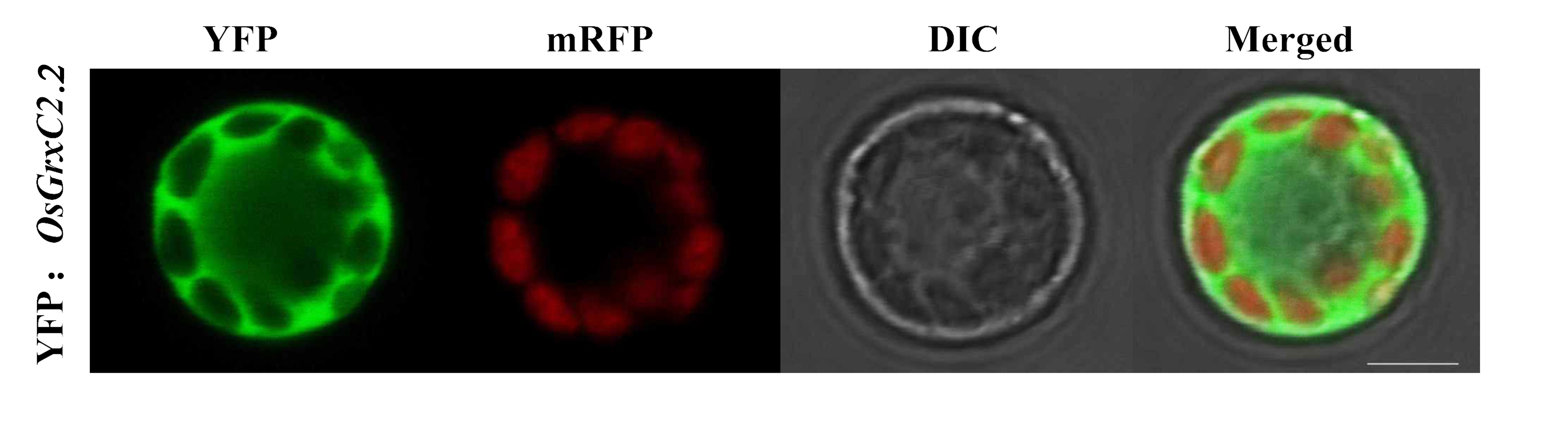

Supplement: FIGURE S1 — Subcellular localization of the OsGrxC2.2 protein. [file Image_1.JPEG]

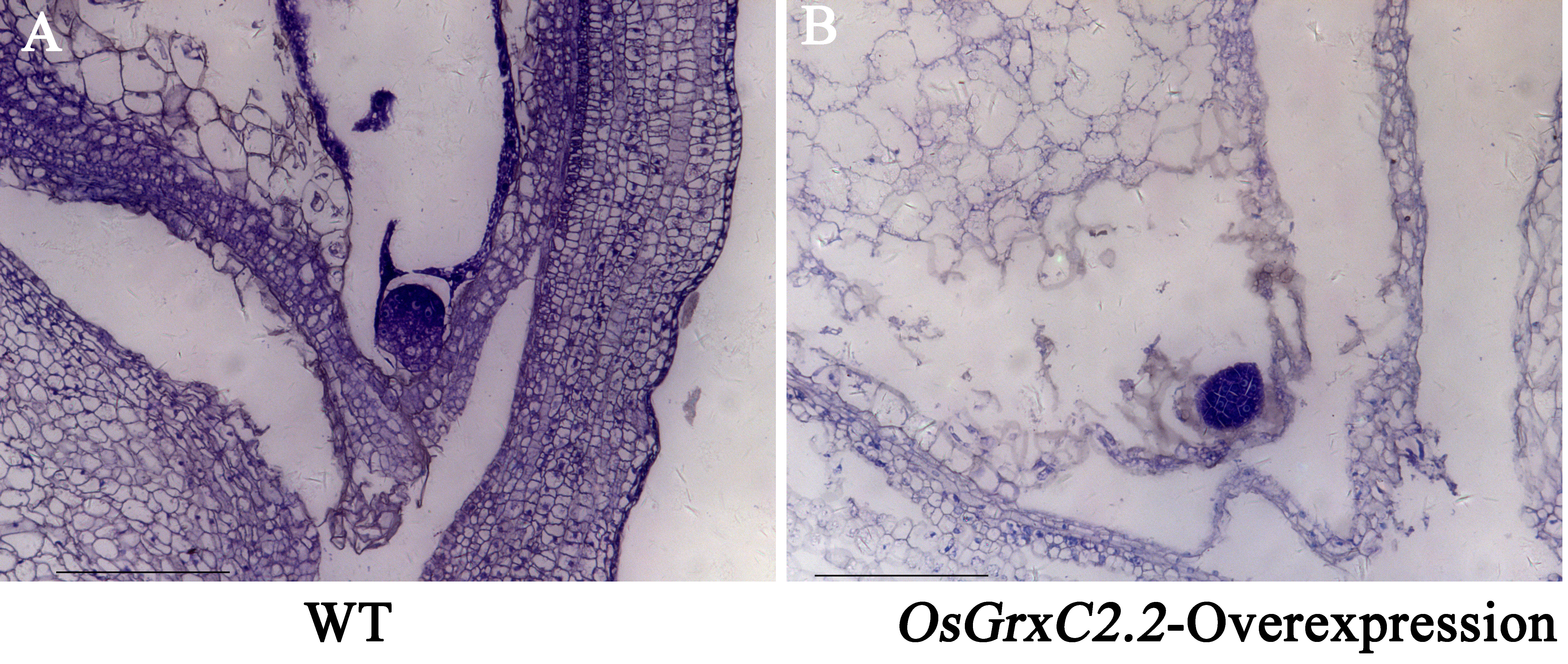

Supplement: FIGURE S2 — Median longitudinal sections of the WT and OsGrxC2.2-over expression lines embryos at 3 DAP. Developing embryos of WT (A) and representative overexpression line OE11 (B). [file Image_2.JPEG]

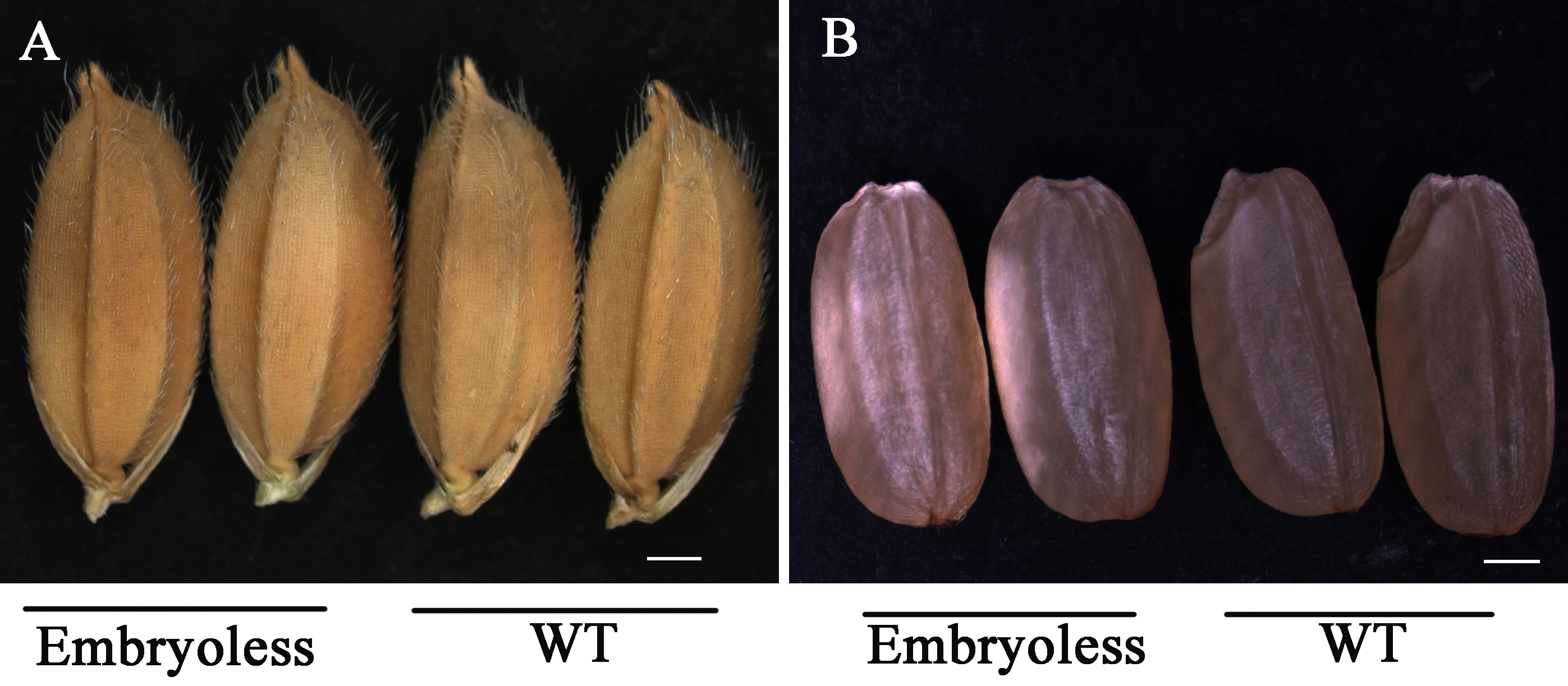

Supplement: FIGURE S3 — Phenotypic analysis of embryoless and WT seeds. (A) Hulled seeds. (B) Shelled embryoless and WT seeds. [file Image_3.JPEG]

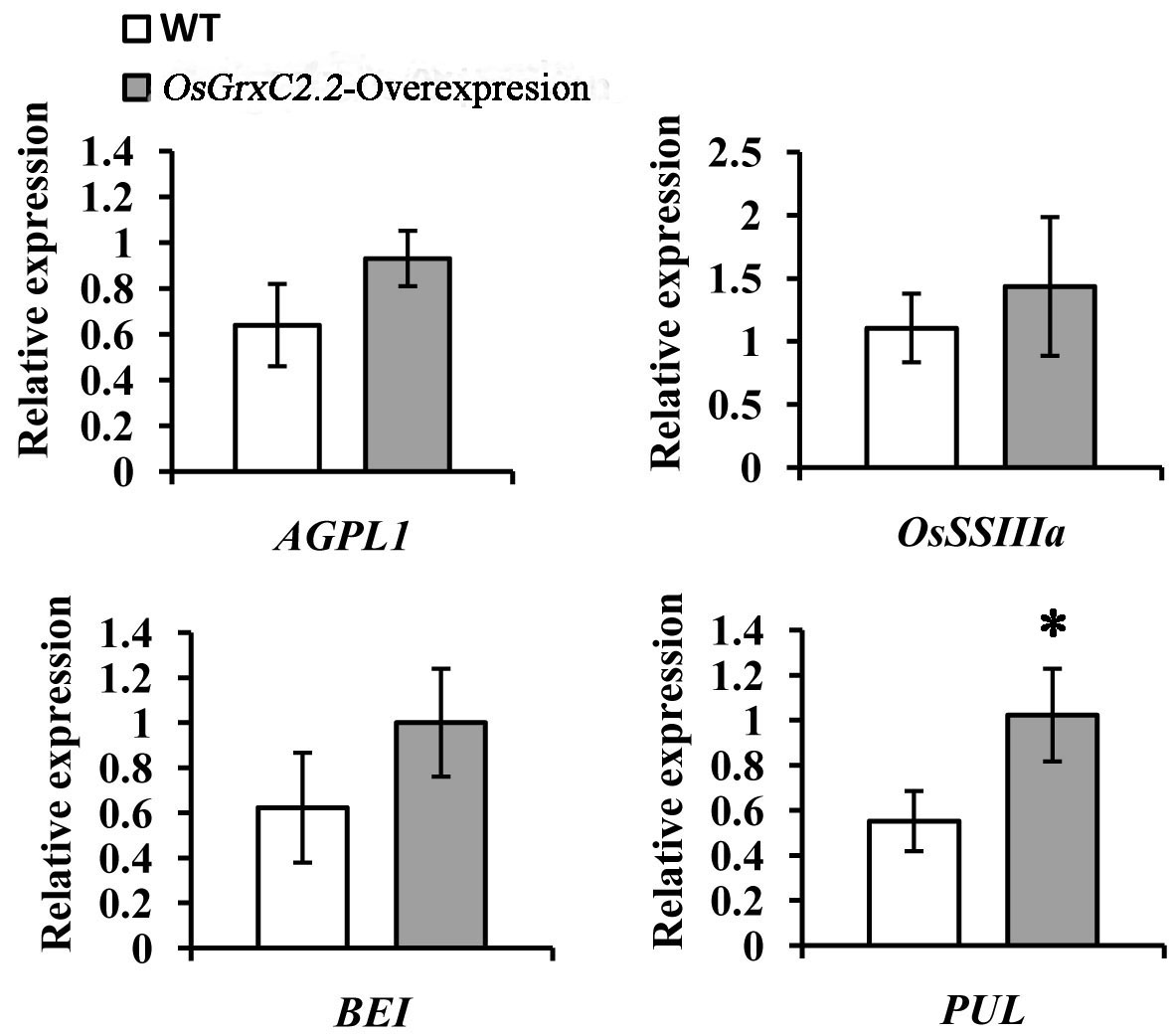

Supplement: FIGURE S4 — Analysis of endosperm development maker genes in WT and OsGrxC2.2-overexpression lines. Expression levels of genes involved in starch biosynthesis obtained via qRT-PCR analyses of AGPL1, OsSSIIa, BE1, and PUL from 10 DAP seeds of WT and OsGrxC2.2-overexpression lines. The y-axis represents the gene expression relative to the OsActin1 transcript level. Results are the averages of three independent experiments. [file Image_4.JPEG]

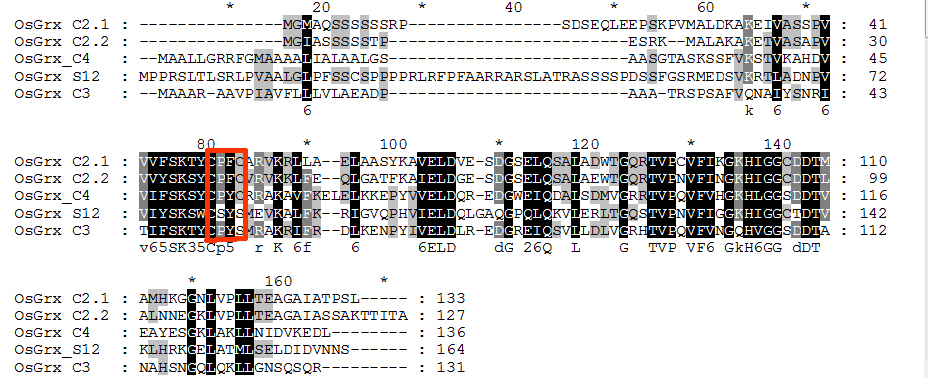

Supplement: FIGURE S5 — Amino acid sequence alignment between OsGrxC2.2 and other class I Grx sequences in rice using ClustalW software. [file Image_5.JPEG]
