# Supplementary material for: Overexpression of a CPYC-Type Glutaredoxin, OsGrxC2.2, Causes Abnormal Embryos and an Increased Grain Weight in Rice
Source: Front Plant Sci. 2019 Jun 27;10:848. doi: 10.3389/fpls.2019.00848 (PMC6610441; doi:10.3389/fpls.2019.00848)
Supplement: TABLE S1 — Primer sequences used in this study. [file Table_1.DOCX]

| **Primer name** | **purpose** | **Sequence (5’-3’)** |
| --- | --- | --- |
| AGPL1 qF | qRT-PCR | GGAAGACGGATGATCGAGAAAG |
| AGPL1 qR |  | CACATGAGATGCACCAACGA |
| OsSSIIIa qF |  | GCCTGCCCTGGACTACATTG |
| OsSSIIIa qR |  | GCAAACATATGTACACGGTTCTGG |
| BEI qF |  | TGGCCATGGAAGAGTTGGC |
| BEI qR |  | CAGAAGCAACTGCTCCACC |
| PUL qF |  | GCTGTCGCTTCTTATGATGCTC |
| PUL qR |  | AAGTGGTCCAGTATAAGCAAACAT |
| ROC1 qR |  | ACAGCAACCCTCAGTAGTAG |
| ROC1 qR |  | CCAACAAGCAACAACCACAAGT |
| ROC2 qF |  | ATCAGTGGGAAGTCAAGAAC |
| ROC2 qR |  | GAGAGGCGAACATGAAGAGG |
| ROC3 qF |  | GAGCAACATGGGATCAGACAC |
| ROC3 qR |  | TGAGTAGCTGTTAGTGTTGG |
| HAZ1 qF |  | GAAGACTGGCAGGTGAAACTC |
| HAZ1 qR |  | GTCCTAATTCAAGGTAGTACAG |
| RAmyl1A qF |  | CAAAGATTGGACCAAGATACG |
| RAmyl1A qR |  | GAAGTACTTCGTGGACAATTG |
| HB1 qF |  | GTGCAGGCAAGGAGATAAGAG |
| HB1 qR |  | CTGAAGCCGTGAAGACTCCTTC |
| HB2 qF |  | TGCTCGCAGTCATCGTCGTTG |
| HB2 qR |  | CCAGATCAAATTAGTGCAAAAC |
| HB3 qF |  | CAGGCTTCCGTGTGATACCA |
| HB3 qR |  | AAGTGTGCGTGTCGCAGATG |
| OsSCR qF |  | CGATGGATACACGCTTATTGAG |
| OsSCR qR |  | GATCAAGTGATACTTCAGCTC |
| OsPNH1 qF |  | CCACTGGGACGAACGGAAC |
| OsPNH1 qR |  | GATACAACAACTATTATACATGC |
| OSH1 qF |  | ACGAGATGCAGTTCGTGATGATG |
| OSH1 qR |  | TCGAACGATCAGCAAATTATATAATC |
| OsActin1 qF |  | GGAACTGGTATGGTCAAGGCT |
| OsActin1 qR |  | ACACGGAGCTCGTTGTAGAAG |
| *OsGrxC2pro* F  *OsGrxC2pro* R  *OsGrxC2-*PHQSN1 F  *OsGrxC2-*PHQSN1 R  *OsGrxC2-*EYFP F  *OsGrxC2-*EYFP R  *OsGrxC2-*PET28a F  *OsGrxC2-*PET28a R | Vector construction | gagctcCTACTCAATTCACAACCTCGCATG  gtcgacCTCTGCGGTGTGACGTCG  cgggatccATGGGAATCGCCTCCTCC  cgggatccCTATGCGGTGATTGTCGTCTTTG  ccgaattcATGGGAATCGCCTCCTCC  cgggatccCTATGCGGTGATTGTCGTCTTTG  ggatccATGGGAATCGCCTCCTCC  ctcgagCTATGCGGTGATTGTCGTCTTTG |
|  |  |  |
|  |  |  |
